# Latent variable mixture modelling and individual treatment prediction

**DOI:** 10.1016/j.brat.2019.103505

**Published:** 2020-01

**Authors:** Rob Saunders, Joshua E.J. Buckman, Stephen Pilling

**Affiliations:** Research Department of Clinical, Educational and Health Psychology, University College London, Gower Street, London, WC1E 7HB, UK

**Keywords:** Latent profile analysis, Psychotherapy, IAPT, Precision medicine, Treatment outcomes

## Abstract

Understanding which groups of patients are more or less likely to benefit from specific treatments has important implications for healthcare. Many personalised medicine approaches in mental health employ variable-centred approaches to predicting treatment response, yet person-centred approaches that identify clinical profiles of patients can provide information on the likelihood of a range of important outcomes. In this paper, we discuss the use of latent variable mixture modelling and demonstrate its use in the application of a patient profiling algorithm using routinely collected patient data to predict outcomes from psychological treatments. This validation study analysed data from two services, which included n = 44,905 patients entering treatment. There were different patterns of reliable recovery, improvement and clinical deterioration from therapy, across the eight profiles which were consistent over time. Outcomes varied between different types of therapy within the profiles: there were significantly higher odds of reliable recovery with High Intensity therapies in two profiles (32.5% of patients) and of reliable improvement in three profiles (32.2% of patients) compared with Low Intensity treatments. In three profiles (37.4% of patients) reliable recovery was significantly more likely if patients had CBT vs Counselling. The developments and potential application of latent variable mixture approaches are further discussed.

## Highlights

• LVMM can identify clinical phenotypes or “profiles” using routine patient data.
• Profiles can be used to predict multiple important clinical outcomes.
• These profiles provide robust probabilities of outcome to psychological treatment.
• People in different profiles benefit more from some interventions than others.
• The profiles could be used to inform personalised treatment recommendations.

## 1 Introduction

The ‘personalised medicine’ initiative aims to improve treatment outcomes and healthcare efficiency by identifying the intervention(s) which have the greatest probability of optimizing outcomes for each individual patient (Khoury & Galea, 2016). In the treatment of mental health conditions such as depression and anxiety, there are large individual variations in treatment outcomes; around 50% of patients recover following treatment but 50% do not, despite having the same diagnosis and even when they have had the same treatment as those who did respond (Clark, 2018). The identification of groups of patients, based on information provided prior to starting treatment (such as demographic and self-reported symptom data), can help understand the likely different response to treatments between these groups, and support effective decision making by stratifying care (Saunders, Cape, Fearon, & Pilling, 2016). This person-centred approach assumes there are some common characteristics within a group of individuals that make them more likely to respond to a given treatment compared to groups with a different set of common characteristics, i.e. those with a different clinical profile.

Tailored approaches which personalise the treatment delivered have led to improvements in the selection and delivery of care in a number of physical health settings. For example, the identification of patient sub-groups with specific BRAC-1 gene mutations has helped to improve outcomes for patients with breast and ovarian cancers (Schwaederle et al., 2016) informing clinical decisions about specific chemotherapies (Kennedy, Quinn, Mullan, Johnston, & Harkin, 2004). Pharmacogenetic testing has been used to inform the selection of drugs and doses for patients with cystic fibrosis by basing treatment regimen decisions on the likelihood of each individual patient achieving optimal outcomes or reducing the risk of harm (Ashley, 2015; Pirmohamed, 2014).

Until recently personalised approaches to medicine have been limited to the treatment of physical health conditions, despite the potential benefits to patients with mental health conditions (Ozomaro et al., 2013). A number of research groups have recently developed personalised models and approaches for mental health, with the greatest focus on treatment of depression. Early studies in this area predominantly aimed to determine the benefit of starting a pharmacological treatment (e.g. an antidepressant such as Escitalopram) compared to an alternative (e.g. another antidepressant, placebo or a psychological treatment such as cognitive behaviour therapy: CBT) (e.g. Chekroud et al., 2016; DeRubeis et al., 2014; Webb et al., 2018). More recently however, there have been a number of efforts to compare psychotherapies: CBT compared to psychodynamic psychotherapy for depression (Cohen, Kim, Van, Dekker, & Driessen, 2019), and CBT compared to Interpersonal Psychotherapy (IPT) (Huibers et al., 2015), as well as investigating the advantage of antidepressant medications or continuation CBT in the context of relapse prevention (Vittengl, Anna Clark, Thase, & Jarrett, 2017).

These treatment selection models have demonstrated the potential advantage for any given patient in receiving one specific type of treatment over another, but the inclusion of the outcome within the development of their predictive models means they are restricted to predicting just one outcome (Kent, Stochkendahl, Christensen, & Kongsted, 2015). This may be exactly as required in certain clinical situations including clinical trials, but for many areas of clinical practice there may be a need to assess the likelihood of more than one potential outcome (which may or may not be related) when making treatment decisions, such as reduction in clinical symptoms alongside the risk of particular adverse effects of a given treatment. Traditional variable-centred approaches focus on how variables are related to one another, and are therefore more limited in informing on the prediction of multiple (potentially contradictory) outcomes, whereas person-centred approaches, such as sub-group identification methods, explore how variables group together within individuals. Identifying subgroups (or clinical profiles) which can be used to inform clinical decision making, may provide an optimum method of supporting treatment selection decisions, particularly in the context of competing or contradictory clinical needs or outcomes (e.g. remission against risk of harm).

### 1.1 Subgroup identification and personalised medicine

The categorisation of individuals into subtypes is often desirable when describing complex relationships between patient factors and outcomes (Herzberg & Roth, 2006); diagnostic manuals subtype patients based on symptoms to inform likely prognosis and treatment decisions (Merz & Roesch, 2011). Personalised treatment recommendations based on subtypes of patients might require a two-phased approach, whereby the identification of sub-groups or clinical profiles of individuals is conducted in the first phase, before the association between identified profiles and outcomes with a number of interventions is explored in a second phase. This data-driven approach does not work backwards from an outcome of interest in the way that variable-centred approaches (e.g. those based on regression models) do, but instead explores the inter-relationship between indicator variables, such as a patient-related characteristics (e.g. age, gender, baseline symptom severity) (Howard & Hoffman, 2018).

One goal of these profiling approaches is, therefore, to identify ‘clinical phenotypes’ that can be used to inform treatment decisions and that have clinical validity (Calfee et al., 2014). Clinical decision-making theories suggest that clinicians may compare new patients to existing ‘prototypes’, that is groups of similar patients the clinician has already treated or patients that ‘typify’ a group of patients with a specific problem or disorder that they are familiar with (Garb, 2005). Profiling methods could create more objective ‘prototypes’ of patients seeking treatment and if linked to the likelihood of specific outcomes then they could be used to inform treatment selection decisions.

There are other potential advantages to these approaches over variable-centred approaches: the personalised medicine studies in mental health discussed above, have all used one or more supervised machine learning algorithm(s) to develop models that can predict outcomes for each individual, i.e. variable-centred approaches. However, the choice of variables selected by such models and the way the models handle the variables to produce predictions can be difficult to understand and interpret, and have been criticized as being ‘opaque’ (Dwyer, Falkai, & Koutsouleris, 2018), which could potentially reduce the trust clinicians have in the models, and thereby reducing their utility (Hart & Wyatt, 1990). Further, one of the concerns expressed in the literature on personalised treatment outcome predictions in mental health is that many of the models developed and tested have yet to be confirmed as useful ‘out-of-sample’; there is considerable doubt about their performance for patients whose data were not used to develop the treatment selection model. In particular, there is doubt about the model performance when tested prospectively in different settings or contexts (e.g. not in clinical trials) to those which were used to gather data used to build the models (Chekroud & Koutsouleris, 2018). Initiatives to test a number of the approaches discussed above and consider their ‘out-of-sample’ performance have begun (Cohen et al., 2018) but full results are not yet available. Identifying clinical phenotypes which are not reliant on outcomes, are robust across settings, and are predictive of a number of treatment outcomes may provide further value over and above variable-centred analyses such as the supervised machine learning algorithms based on regression models.

### 1.2 Latent variable mixture modelling (LVMM)

One way of identifying clinical profiles within a sample in a person-centred way is provided by a family of approaches called latent variable mixture modelling (LVMM) (Berlin, Williams, & Parra, 2014), which includes latent class analysis (LCA) for categorical indicator variables or latent profile analysis (LPA) for continuous indicator variables (Goodman, 1974; Hagenaars & McCutcheon, 2002; Lazarsfield & Henry, 1968). These clustering approaches are based on probability theory and are used to identify homogeneous sub-groups within samples (Finch & Bronk, 2011; Merz & Roesch, 2011). In LVMMs, the probability of the specific pattern of responses y, P(Y = y), is a weighted average of the C class-specific probabilities P(Y = y|X = x); expressed as:

$$P\left(Y=y\right)=\overset{C}{\underset{x=1}{\sum }}P\left(X=x\right)P\left(Y=y|X=x\right)$$

where P(X = x) is the proportion of individuals in Latent Class x. Groups of individuals with a similar pattern of responses or characteristics are identified to determine statistically differing groups within the dataset. Each individual case can be classified into a subgroup (referred to as the individual's latent class or latent profile) from their observed pattern of responses (e.g. to questionnaire items, presence of particular symptoms, or demographic characteristics). The inter-item relationship is explained by the presence of a ‘latent’ unknown subgroup (e.g. the profile). Individual differences in the observed item response patterns are explained by differences in latent class/profile membership. Compared to alternative clustering methods such as K-means clustering (Cox, 1957), LVMM approaches benefit from the use of model fit statistics to identify the optimum number of subgroups and a reduced vulnerability to extreme scores or outliers (Magidson & Vermunt, 2002; Schreiber & Pekarik, 2014).

One key additional benefit of latent profile methods for personalised medicine is that they can be used to identify the most likely profile membership of individuals entering treatment i.e. those that were not used to develop the profiling model, by using the previously identified latent classes/profiles. This can be done by calculating the probability of profile membership based the similarity of a new patient's data to those in each identified class/profile, referred to as posterior membership probability. This is calculated using Bayes rule (Bayes, 1763). For categorical variables, this is expressed as:

$$P(X=x|Y=y)=\frac{P\left(X=x\right)P(Y=y|X=x)}{P(Y=y)}$$

For continuous variables, the probability density distribution is used, and expressed as:

$$f\left(x\right)=\frac{1}{\sqrt{2\pi \sigma_{ij}^{2}}}exp\left(\frac{{-(x_{i}-\mu_{ij})}^{2}}{\sigma_{ij}^{2}}\right)$$

where $\sigma_{ij}^{2}$ is the variance of item i within j class, x_i_ is the value of item i and $\mu_{ij}$ the mean of item i for class j.

LVMM approaches allow for the consideration of several, potentially interacting risk-factors, which can be difficult to model in traditional multiple regression approaches (Aiken & West, 1991). The constellation of different factors then can be used to identify individuals with similar phenotypic profiles' (Agrawal, Lynskey, Madden, Bucholz, & Heath, 2007), and LVMM methods have been used to identify subgroups in a range of clinical presentations. These include the identification of phenotypic subgroups (Libon et al., 2014) and cognitive profiles (Scheltens et al., 2016) in Alzheimer's disease, distinct profiles representing different clinical presentations of eating disorders (Wade, Crosby, & Martin, 2006); Personality disorders (Fossati et al., 2001) as well as depression and anxiety disorders (Rosellini & Brown, 2014; Unick, Snowden, & Hastings, 2009).

The application of LVMM can be used beyond clinical and demographic variables, with the potential to identify phenotypes using biomarkers and genetic data (Conley, 2017). Such approaches have been applied to identify genetic subgroups of conditions such as Crohn's Disease (Cleynen et al., 2010), but at present analyses have not incorporated genetic information in mental health subgrouping.

### 1.3 LVMM and treatment prediction

Although there have been a number of previous studies identifying clinical profiles in mental health samples few have explored the ability of the identified profiles to predict treatment outcomes. This is despite the potential benefits of this person-centred approach being able to compare across multiple outcomes. More recently latent profile analysis has been used to both identify statistically different groups of patients attending psychological treatment for common mental disorders and differences in outcomes to treatment between these profiles (Saunders et al., 2016). This analysis identified eight distinct profiles in a dataset of over 16,000 patients, and explored the outcomes between these profiles. The study found considerable differences between profiles over a range of outcomes, for example the likelihood of recovery ranged from 15% to 75% between profiles, and the likelihood of deterioration from 5% to 20%. Within these profiles there were further differences in outcomes when the same group of patients received either low intensity interventions (for example facilitator-supported self-help materials) or high intensity (more formal face-to-face treatments such as CBT) interventions, and as such the method had the potential to inform treatment selection decisions.

The findings of this previous study have the potential to inform treatment decisions, but require out-of-sample validation (Chekroud & Koutsouleris, 2018; Cohen et al., 2018) and replication of the likely treatment outcomes by profile to further demonstrate their utility to psychological treatment services. For example, differences in clinical presentations to psychological treatment services may have resulted in different clinical phenotypes being more prevalent, and changes to clinical practice may have resulted in differences in the likelihood of outcomes from the previously identified profiles. The utility of the profiles would be further enhanced if they were able to further predict the likelihood of treatment outcomes between different types of treatment modality, beyond just intensity of treatment as presented in Saunders et al. (2016).

The aims of this article are therefore to explore the stability of treatment outcomes for the previously identified profiles, to assess whether their predictive ability is replicated out-of-sample, and to test whether they can further differentiate differences in outcome between modalities of psychological treatment. This validation will be conducted in a dataset of approximately 18,500 patients receiving psychological treatment for common mental disorders in UK mental health services.

## 2 Example of LVMM in treatment prediction

### 2.1 Data description

The dataset used in this analysis was provided from two Improving Access to Psychology Therapies (IAPT) services in London. IAPT services were initiated in England in 2008 to increase the availability of evidence-based psychological treatments for depression and anxiety disorders. Treatment is typically delivered following a stepped-care model whereby low intensity (LI) therapies (e.g. facilitator led/guided self-help, or computerized-CBT) might be offered for patients with more mild to moderate presentations and high intensity (HI) therapies (e.g. CBT, interpersonal therapy (IPT) or counselling) might be offered for those with more moderately severe presentations (Buckman, Saunders, Fearon, Leibowitz, & Pilling, 2018). For further information on IAPT services see Clark (2018). The dataset used in this paper was provided as part of a wider service improvement project conducted in accordance with the procedures of the host institution and the local services (project reference: 00519 – IAPT).

The dataset for the current analyses included all patients entering treatment between September 2013 and March 2018 and who, as part of a routine outcome measurement programme, provided scores on both the Patient Health Questionnaire 9-items version (PHQ-9; Kroenke, Spitzer, & Williams, 2001) and the Generalised Anxiety Disorder scale 7-items version (GAD-7; Spitzer, Kroenke, Williams, & Löwe, 2006). These patients did not overlap and are therefore independent of the cohort that Saunders et al. (2016) initially used to develop the profiles. Further inclusion criteria were applied to all analyses of treatment outcomes and required that patients were scoring above the clinical threshold for “caseness” on either the Patient Health Questionnaire 9-items (PHQ-9; Kroenke et al., 2001: a score of 10 or above) or the Generalised Anxiety Disorder scale 7-items (GAD-7; Spitzer et al., 2006: a score of eight or above) at baseline assessment. In addition, only patients who provided an initial, and at least one further symptom severity score on each scale were included in this analysis, and therefore had two or more treatment sessions, to allow for the calculation of treatment outcomes. These criteria were adopted in order to calculate the outcome metrics used to evaluate all IAPT services nationwide (NHS Digital, 2016). Finally, any patient with missing data on three or more of the key variables used in the latent profile algorithm (see below Table 1) was excluded from analyses (approximately 3.7% of the sample).

**Table 1:** Included indicators in the latent profile algorithm.

Variable name	Description	Variable type
Depression severity	Patient Health Questionnaire (PHQ-9) at referral (Kroenke et al., 2001)	Continuous
Anxiety severity	Generalised Anxiety Disorder Scale (GAD-7) score at referral (Spitzer et al., 2006)	Continuous
Functional impairment	Work and Social adjustment Scale (W&SAS) score at referral (Mundt, Marks, Shear, & Greist, 2002)	Continuous
Phobia self-rating	Caseness for phobia was defined as a score of 4 or higher on any one of the three phobia items (IAPT, 2011).	Dichotomous
Age at referral	Age of patient	Continuous
Gender	‘Male’ or ‘female’	Dichotomous
Medication prescription status	‘Prescribed’ or ‘not prescribed’ psychotropic medication at referral.	Dichotomous
Welfare status	‘Receiving benefits’ as defined by the IAPT employment status variable	Dichotomous
Ethnic group	‘White’ or ‘non-white’ ethnic group	Dichotomous

### 2.2 Variables

The latent profiles are generated using nine patient level characteristics that are collected as part of the IAPT minimum dataset (MDS) and are therefore available to all local IAPT services, and these are presented in Table 1. Several studies have reported on a number of other factors that might help give more accurate predictions of treatment outcomes in IAPT, for example expectancy of benefit with treatment (Delgadillo, Moreea, & Lutz, 2016) and attentional control (Buckman, Naismith, et al., 2018) are related to symptomatic improvement in treatment, and alcohol misuse was associated with treatment attrition (Buckman, Naismith, et al., 2018). However, as these are not routinely used in all IAPT services they were not available for the present analyses.

Formal diagnoses are not routinely collected in IAPT and instead information about the patient's ‘problem descriptor’ is used in order to match patients to evidence-based treatments for certain disorders (e.g. trauma-focused CBT is one of the treatments recommended for post-traumatic stress disorder) (Buckman, Naismith, et al., 2018; Clark, 2018). However, the completion of problem descriptor was initially poor with up to 70% missing in of IAPT patients nationally, but has rapidly improved in completion in more recent years (Clark et al., 2018; Clark, 2018). Although problem descriptors/diagnoses may be associated with decisions about the type and intensity of treatment, for the current study they were not considered in analyses due to concerns over data quality and the significant missingness of this variable in routine care.

The outcomes of interest in this analysis include two of the main outcomes used in NHS Digital's annual IAPT national reports (NHS Digital, 2016, NHS Digital, 2017), reliable improvement and reliable recovery, and these were supplemented by an additional outcome of interest: clinical deterioration. These outcomes are defined below:

• Reliable improvement: A reduction in symptom scores above the error of measurement on either scale (a score of 6 or more on the PHQ-9 and 4 or more on the GAD-7).
• Reliable recovery: Scoring above the clinical caseness cut-off at first assessment on either the PHQ-9 (10 or higher) and/or the GAD-7 (8 or higher), and then scoring below caseness on both measures at the end of treatment, as well as meeting criteria for reliable improvement as defined above.
• Clinical deterioration: A reliable increase in symptom scores above the error of measurement for either the PHQ-9 or GAD-7 (same thresholds as above).

In addition, IAPT's primary outcome of interest, recovery, (defined as scoring above caseness before treatment and below caseness on both measures after treatment, without the need for reliable improvement), as well as treatment attrition (defined as either ‘dropping out of treatment’ or when recorded as ‘declining treatment’ after more than two treatment sessions) were also explored. These outcomes are presented in the supplementary materials only to reduce the amount of information presented in the main article.

### 2.3 Interventions

Data relating to the types of IAPT interventions received by patients were used to explore differences in treatment outcomes between the profiles. Due to the stepped care model delivered in IAPT services some patients may receive more than one type of treatment during an episode of care. For example, individuals who start treatment with LI but show little improvement may be stepped up to an HI treatment during an episode of care, and those who require more than one type of treatment may attend groups or workshops in addition to other interventions (e.g. focused on relapse prevention or on sleep problems).

For analyses comparing outcomes between different intensities (LI vs HI) of treatment, only patients who received treatment at one intensity without being stepped up or down between LI and HI during their episode of care were included in the analyses. When comparing different types of treatment modality (e.g. CBT vs Counselling), we adopted a similar rule to that used by the national reporting of IAPT outcomes (NHS Digital, 2016) that the last treatment received was assigned to the episode of care, but we further specified that the last treatment had to be received for over half of the total treatment sessions and that only one intensity of treatment was received through their episode of care (no step-ups were included). Episodes of care that did not meet these criteria were not included in analyses comparing treatment modalities.

### 2.4 Demonstration latent profiling model

This analysis uses previously identified latent profiles which have been described in detail by Saunders et al. (2016) and validated in a number of IAPT services. A brief description of the eight latent profiles (LPs), using relative descriptors for the sample population, is provided below and the profiles will be referred to by number (e.g. “LP1”) throughout this article:

LP1: Members of this profile are younger than the average of the patients attending the services and have lower symptom severity scores. They are less likely to be cases on the phobia scales, have above average levels of personal functioning and are less likely to be receiving welfare benefits or prescribed psychotropic medication. LP1 include 17.6% of patients entering treatment at the IAPT services.

LP2: This group are also younger than the average for the sample, they report similar levels of depression and anxiety severity to the average for all patients but report above average levels of personal functioning. Patients in this profile are unlikely to be receiving benefits or to be prescribed medication, and are unlikely to be cases on the IAPT phobias scales. This is the most prevalent profile attending the IAPT services (24.1% of patients).

LP3: This profile has the highest mean age and the lowest mean severity scores, suggesting a group of older patients seeking treatment for less severe common mental health disorders. The likelihood of psychotropic medication prescription or receiving benefits is low, and this profile has a low proportion of patients from non-white ethnic groups. LP3 are the least common profile of patients attending the services (3.04%).

LP4: Members of this profile are also considerably older than most people attending the IAPT services, and are similar to LP3 with regard to medication prescription status and ethnicity. However, LP4 patients report moderate levels of depression and anxiety severity and more significant functional impairment. This profile includes 4.92% of patients.

LP5: This profile is made up of those with above average age, PHQ-9 and GAD-7 scores, as well as a higher likelihood of medication prescription, higher incidence of phobia caseness, and are more likely to be in receipt of welfare benefits. LP5 comprises 9.58% of patients entering treatment.

LP6: This profile stands out as having the most noticeable difference between PHQ-9 and GAD-7 scores at assessment, with higher levels of severity for depression than for anxiety. The proportion of patients receiving welfare benefits and prescribed psychotropic medication is higher than the sample average, and it includes 8.21% of patients.

LP7: Members of this profile report the most severe depression and anxiety symptoms at assessment compared to other profiles. They are also older than the average IAPT patient and have a higher likelihood of being prescribed medication and receiving welfare benefits. Nearly 10% of patients were identified as members of LP7.

LP8: This profile contains the highest proportion of patients from non-white ethnic groups, has the highest proportion of female members, and has a below average mean age, lower than that of all the other profiles. This profile has above average severity of depression and anxiety, but has average levels of benefit receipt and medication prescription. This is the second most prevalent profile with 22.91% of patients identified as members of LP8.

### 2.5 Analysis

The latent profile allocation algorithm (Saunders et al., 2016) was applied to the dataset and each patient was allocated to the latent profile to which they had the highest probability of profile membership. In addition, the profile to which they had the second highest probability of membership was also calculated.

A comparison of the baseline and endpoint characteristics between the current sample and the sample used by Saunders et al. (2016) is presented in Table 2. Comparison suggests that patients in the new sample were more likely to be of non-white ethnicities, prescribed medication and less likely to be in receipt of benefits. Pre-treatment levels of depression and anxiety severity were similar between samples. The current sample had a higher mean number of treatment sessions, lower depression and anxiety symptoms post-treatment compared to the sample used in Saunders et al. (2016), as well as higher post-treatment personal functioning scores, which is in line with national trends in outcomes from IAPT services (NHS Digital, 2019). The descriptive statistics of each profile alongside the full sample of patients who entered treatment for the current dataset is presented in Appendix A (Table A1).

**Table 2:** Comparison of samples: Current analysis and Saunders et al. (2016).

	Current sample		Previous sample (Saunders et al., 2016)			
Baseline characteristics	n = 44095		n = 16636			
	Mean	SD	Mean	SD	t	p
Age - Mean (SD)	37.56	14.36	37.9	13.36	2.651	0.008
PHQ-9 - Mean (SD)	13.8	6.46	13.85	6.67	0.843	0.399
GAD-7 - Mean (SD)	12.35	5.43	12.35	5.51	0	1.0
WSAS - Mean (SD)	17.17	9.2	17.85	9.69	8.004	<0.001
	N	%	N	%	z	p
Gender - n(%) female	29561	67%	10793	66%	2.82	0.005
Ethnic Group - n (%) Non-White	11242	30%	3151	22%	18.34	<0.001
Medication prescribed - n (%) prescribed	21310	51%	5802	39%	26.84	<0.001
Welfare status - n (%) on benefits	11230	26%	3834	28%	−5.36	<0.001
Phobia Self-rating - n (%) phobia	20992	52%	7592	51%	3.22	0.001
**Endpoint Characteristics**	**n = 18514**		**n = 10693**			
	Mean	SD	Mean	SD	t	p
Number of treatment sessions	9.35	5.86	6.87	5.68	−35.53	<0.001
PHQ-9 - Mean (SD)	9.52	6.40	10.44	6.92	11.25	<0.001
GAD-7 - Mean (SD)	8.63	5.60	9.23	5.91	8.52	<0.001
WSAS - Mean (SD)	13.27	9.05	14.62	10.20	10.27	<0.001
	N	%	N	%	z	p
Recovered	8432	45.54	4283	40.05	−9.12	<0.001
Reliably Improved	12053	65.74	7041	65.85	1.29	0.197

Note: Comparison of baseline and endpoint characteristics between samples. P-values for t and z tests are presented.

The prevalence of the latent profiles was explored in the dataset to observe possible differences in the distribution of the profiles over time. Next, the proportion achieving each outcome following IAPT delivered psychological treatment was compared across the latent profiles, with a number of clinical outcomes included (as defined above). Thirdly, differences between different IAPT delivered treatment types was compared to present areas where the profiles could be used to make refined personalised treatment predictions. All analyses were conducted using Stata15 (StataCorp, 2017).

## 3 Using latent profiles for prediction

### 3.1 Distribution of profiles

**Fig. 1:**
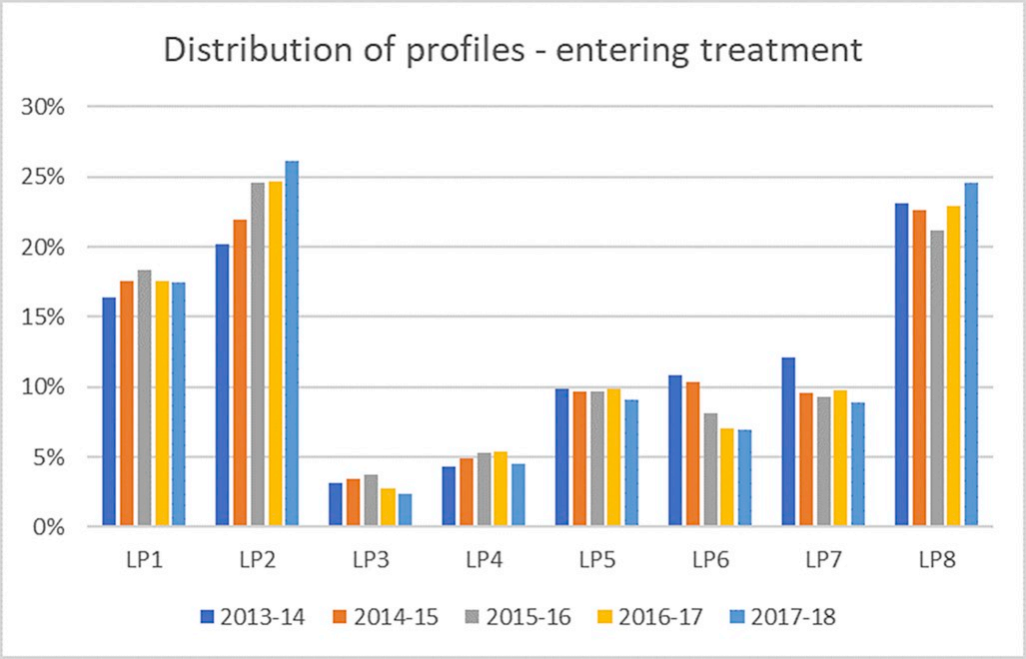
Prevalence of profile by year.

A total of n = 44,095 patients met initial inclusion criteria and entered a course of treatment at one of the two IAPT services. The distribution of profiles by year is presented in Fig. 1 and shows that the proportion of cases assigned to each profile was relatively stable over time (further information in Table A2; Appendix A). However, there were a few differences of note: The proportions of cases assigned to LP6 and LP7 decreased over the five years both by approximately 3.8%, whereas the proportion of patients assigned to LP2 appears to have increased each year, with a 6.4% increase over the five years. It is worth noting that the profiles and outcomes of the Saunders et al. (2016) study were presented to the IAPT services in 2014, indicating that LP7 were at high risk of poor response to IAPT-delivered treatment and that LP6 were at significant risk of deterioration. Therefore assessments may have been able to identify those in these profiles and consider referring them on to other services that might potentially be better suited to offer care for the particular needs of those patients.

**Fig. 2:**
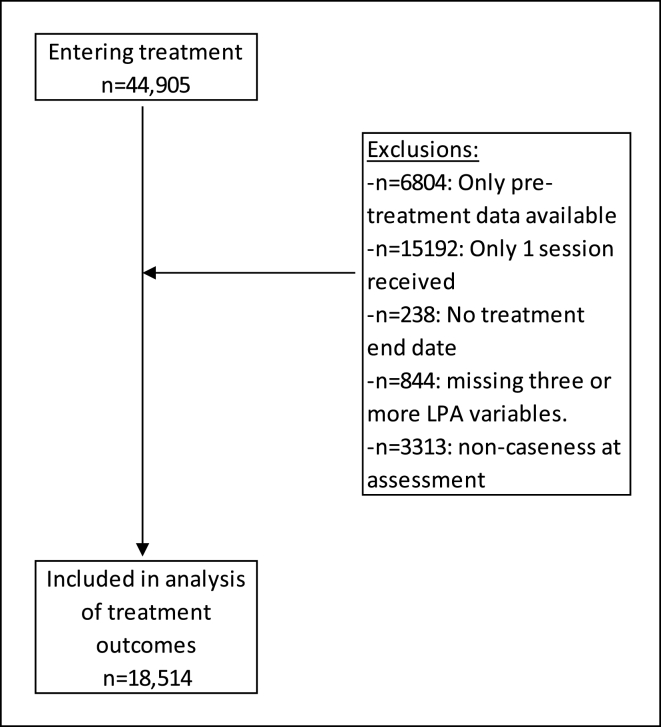
Patient flow diagram for inclusion.

The flow of patients through the analyses are displayed in Fig. 2, although we started with a sample of approximately 45,000 participants, after applying inclusion criteria less than half that number (18,514) were available for the main analyses. The majority of excluded patients received one session of treatment only, which may have been for advice or consultation. The distribution of profiles per year for those who received a course of treatment (had two or more treatment sessions) and were in caseness at assessment is presented in Table A3 and Figure A1 (Appendix A). The distribution of profiles across years for patients receiving a course of treatment appears very similar to those for all patients who entered treatment (Fig. 1), except LP1 and LP3 who are significantly less prevalent owing to the low likelihood of caseness for these patients due to low symptom scale scores on average.

### 3.2 Treatment outcomes

**Fig. 3:**
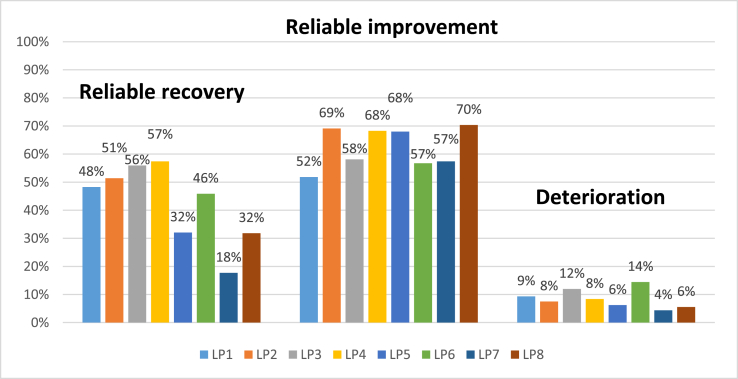
Comparison of the probability of recovery and reliable recovery across profiles.

The original development study that identified the latent profiles found a significant variation in outcomes between profiles (Saunders et al., 2016), and the current analysis on a newer dataset replicated these findings. Fig. 3 displays the proportion of patients meeting the three main outcomes, and full findings including the recovery and attrition outcomes are presented in Appendix B. Patients in LP4 had the highest likelihood of reliable recovery and results of logistic regression analysis (presented in Appendix B; Table B3) showed that the odds of reliable recovery were significantly higher for LP4 compared to all other profiles except LP3. Lowest likelihood of reliable recovery was found for LP7, who were significantly less likely to report reliable recovery than all other profiles, and the odds of this outcome were 6.26 higher for LP4 compared to LP7 (Odds ratio (OR) and 95% Confidence Interval (CI)) = 6.26(5.22–7.52).

There was less variation in the probability of achieving reliable improvement across profiles, and it is of note that the profiles with some of the higher probabilities of reliable recovery did not have higher than average likelihoods of reliable improvement (LP1 and LP3). This is likely due to the degree of baseline dependency in the outcomes, i.e. those with high scores at baseline have more chance of meeting criteria for reliable change as they have more available points on the symptom scales to change on, whereas those with lower scores are more likely to score below the cut-off for caseness than those that score very high on the measures (e.g. Button et al., 2015). However, the profile with the highest initial severity scores (LP7) was not the profile with the highest probability of reliable improvement, which suggests that other factors within the profile are influencing outcomes beyond the effect of baseline symptom severity. Instead LP8 showed the highest likelihood of reliable improvement (70%) and the odds of this outcome were 2.2 times higher compared to LP1: OR (95%CI) = 2.2(1.95–2.49) (Appendix B; Table B4).

The analysis comparing the rate of deterioration between profiles replicated the previous analysis (Saunders et al., 2016) and again showed that LP6, followed by LP3 had the highest rates of deterioration. The odds of deterioration were 3.67 times higher for LP6 than LP7 (lowest deterioration rate): OR (95%CI) = 3.67(2.75–4.91), and LP6 were significantly more likely to show deterioration than all profiles other than LP3 (Appendix B; Table B5).

As presented in Appendix B (Table B1), the rate of attrition ranged between 13% and 35%, of note, the profiles with higher proportions of older adults had the lowest levels of attrition and the more profiles with more severe/complex presentations pre-treatment were associated with higher levels of attrition.

Sensitivity analyses were conducted to consider the stability of the outcomes in each profile over time by comparing outcomes in each of the five years in which we had complete data. The probability of outcomes in each profile was stable across the years with the exception of one profile (LP3) in which the number of cases were too small to analyse this robustly when splitting data into each of the five financial years for which we had data (see Appendix C; figures C1-C5).

## 4 Treatment modality differences

### 4.1 Low intensity vs high intensity by profile

One potential use of the clinical profiles is to support treatment decision making, and to explore this we first compared the difference in outcomes between patients of each profile who received LI interventions compared to those who received HI. For this analysis, patients who were stepped up or down (and therefore received both intensities of treatment) were excluded.

To control for potential differences within profiles of patients who received LI vs HI treatment, propensity score matching was performed to identify matched controls for LI/HI treatments, using the probability of membership to each profile as eight covariates for matching. This encompasses the distributions of data across the nine variables that the profiles were based upon. We used 1:1 matching and employed a narrow caliper of 0.001, using the PSMATCH2 package in Stata (Leuven & Sianesi, 2003). Matching with replacement was used, allowing the same case to be used as a control on multiple occasions to increase the available sample size. The use of this matching method resulted in a loss of 32.86% of available patients to the analysis. Although this reduced the available sample size, we considered it the most appropriate means of providing unbiased comparisons. It is likely that some of the potential differences in the distribution of probabilities of profile membership were associated with the allocation to HI over LI, and therefore using propensity score matching may result in a more conservative comparison between LI and HI. The number of patients in each profile who received either LI or HI only, as well as those who received both intensities of treatment is presented in Appendix D (Table D1).

Full results are presented in Appendix D and show that HI interventions significantly increased the odds of reliable recovery in two profiles, LP7: OR(95%CI) = 1.50(1.07–2.12), and LP8: OR(95%CI) = 1.33(1.14–1.56), which accounts for 32.5% of patients attending the services (Table D3). When comparing the likelihood of reliable improvement within profiles, it was found that LP1 (OR(95%CI) = 1.40(1.09–1.82) and LP7 (OR(95%CI) = 1.52(1.20–1.92), were more likely to benefit from HI treatments, but LP4 were significantly less likely to benefit from HI (OR = 0.61((0.44–0.84) (Table D4). There were no differences in the rates of clinical deterioration with LI vs HI interventions within any profile, but LP6 (which showed the highest rates of deterioration of all the profiles) were found to have a slightly higher proportion of deterioration from LI treatments than HI (18% vs 14%), OR(95%CI) = 0.73(0.53–1.01) (Table D5).

### 4.2 CBT vs counselling

In the next stage of analysis, we compared differences in outcomes within profiles for specific modalities of HI delivered psychological treatments: CBT and Counselling, for which there was sufficient data to allow for meaningful comparisons. It should be noted that in line with NICE guidance (NICE, 2009) Counselling in IAPT services is indicated for the treatment of depression. We included patients that had received HI interventions only, not those that also had some LI treatment. In addition, the last recorded therapy type had to be either CBT or Counselling and more than 50% of the treatment sessions had to be of that therapy type (CBT or Counselling), i.e. it had to be the patient's ‘main treatment’. The mean number of sessions was very similar (CBT = 11.197, SD = 6.02; Counselling = 11.204, SD = 4.87). Propensity score matching was used to control for patients with similar probabilities of profile membership to all LPs as described above for the LI vs HI comparison using a caliper of 0.001 and 1:1 matching. The matching procedure resulted in a loss of 47% of the initially available sample of patients who received CBT or Counselling, and this reduction compared to the previous analysis (LI vs HI) is likely due to the reduced initial numbers of available patients for the current sub-analysis. Due to low numbers of included patients, LP3 was excluded from these analyses.

The proportion of patients within profiles who achieved outcomes of interest are presented in Appendix E. Results presented in Table E2 show that for LP4, LP5 and LP8 (37.4% of patients entering treatment who had more severe or complex profiles) CBT significantly increased the odds of reliable recovery (LP4 = OR(95%CI) = 2.01(1.30–3.10), p = .002; LP5 = OR(95%CI) = 1.78(1.24–2.58), p = .002; LP8 = OR(95%CI) = 1.57(1.08–2.26), p = .017) relative to Counselling. Reliable improvement was more likely for LP4 and LP5 following CBT vs Counselling (Table E3) (LP4 = OR(95%CI) = 1.66(1.05–2.62), p = .03; LP5 = OR(95%CI) = 2.01(1.39–2.92), p < .001). Higher percentages of patients in LP1 and LP6 achieved reliable recovery after Counselling (LP1 = 55.56%, LP6 = 44.64%) than CBT (LP1 = 50.94%, LP6 = 39.29%) but these were based on relatively small numbers of included patients and were not close to reaching statistical significance (LP1 = OR(95%CI) = 1.20(0.56–2.58), p = .633; LP6 = OR(95%CI) = 1.25(0.73–2.12), p = .417).

When exploring differences in the rates of clinical deterioration it was found that LP4 were at significantly higher risk of deterioration following Counselling (OR(95%CI) = 3.06(1.26–7.42)), and there was a non-significant trend towards the same pattern in LP5 (OR(95%CI) = 1.98(0.94–4.18), however all the analyses of deterioration were based on small numbers of included patients.

## 5 Potential application of ‘secondary profiles’

The above analyses determined each patient's profile membership based on the one profile that they had the highest probability of belonging to, which is standard practice for use of LVMM. However, the role of the probability of membership to other, secondary profiles could potentially be used to further inform outcome prediction. For example, consider the following question: if a member of LP6 shares more characteristics with members of LP2 than members of LP8, by virtue of having a higher probability of membership to LP2 than LP8, then does this change the probability of outcome for patients in this profile (LP6)? A brief demonstration of this ‘sub-profiling’ approach is displayed in this section, and will focus on LP6 as this profile has the highest probability of deterioration yet has a reliable recovery rate approaching 50%.

Table 3 presents the probability of reliable recovery and deterioration for cases whose ‘primary’ profile is LP6 and compares the differences in outcomes dependent on the secondary profile. LP3 was the secondary profile for only three cases, and only four cases had LP7 as their secondary, so these were removed from analysis due to limited numbers. The findings presented in Table 3 for the other secondary profiles suggests that LP6 cases with a secondary profiles LP2 or LP4 have better odds of reliable recovery compared to LP6 cases whose secondary profiles is LP5 or LP8. Turning to deterioration, secondary membership to profile LP2 is associated with a lower likelihood of deterioration whereas secondary membership to profiles LP1, LP4 or LP8 is associated with an increased odds of deterioration for LP6 cases.

**Table 3:** Variation in recovery & deterioration dependent on secondary profile (LP6 cases).

Second profile	n	% reliable recovery	% deterioration
LP1	256	44.53%	16.80%
LP2	961	48.80%	12.85%
LP4	179	45.25%	16.20%
LP5	220	41.36%	14.55%
LP8	156	37.82%	17.31%
All LP6 cases	1779	45.87%	14.42%

In the next analysis, patients from all other profiles except LP6 were grouped based on whether or not their secondary profile was LP6 irrespective of what their primary profile was, and the risk of deterioration was then compared within profiles. LP3 and LP7 were excluded due to limited patients with LP6 as their secondary profile. Table 4 shows that for all profiles, patients whose secondary profile was LP6 had a higher risk of deterioration than those who had a different secondary profile.

**Table 4:** Probability of deterioration within each LP dependent on whether the secondary profile is LP6 or not.

	n	Not LP6		n	LP6	
		Deteriorated	%		Deteriorated	%
LP1	1200	105	8.75%	247	30	12.15%
LP2	3953	272	6.88%	1325	170	12.83%
LP4	915	68	7.43%	182	24	13.19%
LP5	1689	89	5.27%	206	29	14.08%
LP8	4454	227	5.10%	190	30	15.79%

## 6 Discussion

This paper has sought to demonstrate how latent variable mixture modelling approaches can be used to identify clinical profiles of patients receiving treatment for common mental health disorders and used to predict a number of different treatment outcomes. The utility of a previously developed profiling system was presented and validated in an independent sample that differed on a number of important demographic and clinical variables from the original sample, demonstrating replicability of the previous findings. There was a large amount of variation across the profiles in terms of the probability of all outcomes including reliable recovery, reliable improvement and clinical deterioration. We compared the probability of treatment outcomes within profiles between matched patients who received LI vs HI therapies, and found that reliable recovery was significantly more likely for two profiles (LP7 and LP8) following HI treatments. Interestingly, although two profiles had significantly higher odds of reliable improvement following HI (LP1 and LP7), it was also found that one profile significantly benefitted from LI over HI treatments (LP4) which could inform the allocation of resources in psychological treatment services.

Differential outcomes for matched members of the same profile who received either CBT or Counselling (both delivered at HI) were also explored. Findings indicated that for three profiles (LP5, LP7 and LP8) there appeared to be a benefit of CBT over Counselling in terms of achieving reliable recovery, and higher odds of reliable improvement for those in profiles LP4 and LP5, and lower odds of deteriorating for patients in LP4 and LP5 with CBT vs Counselling. This might lead to a recommendation that if patients from LP4 or LP5, choose or are in receipt of Counselling then routine monitoring of symptoms could be helpful to spot potential deterioration early and inform decisions on any adjustments to treatment if necessary.

The uses of information about the probability of membership to other secondary profiles are discussed and a brief demonstration using LP6 shows that the profile to which an individual in LP6 has the next highest probability of membership could be used to refine the prediction of likely treatment outcomes. This might suggest that within profile, an understanding of the association to other latent profiles might improve the prediction of treatment outcomes and potential tailoring of care delivered, particularly when weighing up the probability of recovery with a particular treatment against the probability of deterioration with the same treatment.

### 6.1 Implications

One of the major benefits of person-centred approaches such as LVMM is that multiple outcomes can be compared for individuals with a similar constellation of characteristics (Kent et al., 2015). This can have important benefits over variable-centred models which are restricted to using patient characteristics to predict one outcome at a time. The analyses presented in this paper highlight how the likelihood of different outcomes could be combined to predict the likely benefits of psychological treatments. For example LP6 has a probability of recovery/reliable improvement of over 50% suggesting a likelihood of response similar to the average patient in receipt of IAPT treatment (NHS Digital, 2016). However, the likelihood of deterioration is much higher for this profile of patients (15%) and therefore considering both the probability of recovery along with the relatively high probability of deterioration could be important to determining which treatment might be offered, and the appropriate level of monitoring and review.

This paper has also highlighted the potential for LVMM approaches to inform clinical decisions about the specific modality of psychological treatment. Results indicated that for two of the profiles (LP7 and LP8) there was a significantly higher likelihood of reliable recovery following HI interventions when compared with LI when patients were matched on the probability of profile membership, which might lead to a recommendation that more intensive treatment should be considered the preferred option for these groups of patients. It is interesting that for one profile, LP4, the odds of reliable improvement were significantly better when LI was received instead of HI. In the analyses presented in Saunders et al. (2016), LP4 were also found to have a slightly higher probability of recovery following LI, although this difference was not statistically significant. This might suggest that this group of patients are less likely to respond to more intensive treatment than low intensity treatments, but could also indicate that other factors which differ between the treatments could explain this finding (i.e. residual confounding). One such factor may be the waiting time to start treatment which is typically considerably shorter for LI than HI treatments, this factor could not be assessed in the current analysis but has been found to be related to treatment outcomes in IAPT (Clark et al., 2018).

Further analyses identified that for certain profiles there was a benefit of CBT over Counselling, and this information might be used to inform decisions about potential treatments. LP4, LP5 and LP8 were found to benefit more from CBT treatment than from counselling, with LP4 also more likely to experience a clinical deterioration following Counselling vs CBT. LP5 and LP8 are associated with more complexity with regard to presenting characteristics (higher severity, lower levels of functioning and presence of phobias) and this finding suggests that CBT might be more effective in these circumstances.

The demonstration of the use of conditional probabilities and the identification of an individual's secondary profile (the profile to which they have the second-highest probability of membership) highlights the potential value of this approach to refine the prediction of treatment outcomes using LVMM. As this information is provided as part of LVMM it means that all individuals will have conditional probabilities available and exploration of how best to use this additional information to refine likely predictions of prognosis could further add strength to the latent profiling approach, yet this has not been explored previously.

One further benefit of the person-centred approach presented in this paper is the robustness of the profiles over time with regard to both the distribution of profiles amongst patients entering treatment at the services and the likelihood of outcome for each profile. Analyses demonstrated that the prediction of outcome over time was remarkably stable, validating previous findings, and is an important consideration as current variable-centred approaches have struggled to consistently predict when using different samples from those they were built with (Chekroud & Koutsouleris, 2018). The current analysis used a follow-up of the cohort of that which was used to develop the profiles, and demonstrated that the outcomes for profiles within those services were consistent. Further analyses in different IAPT services, as well as beyond IAPT, are needed to explore the robustness of the profiles in predicting treatment outcomes.

### 6.2 Potential developments

The use of sub-profiling methods, where information about the likelihood of membership to other (secondary) profiles can be used to refine the prediction of treatment outcomes has received little, if any, attention in LVMM. Yet this approach could be adapted to the current profile algorithm as a second stage and further support treatment decisions. The current limitation with the use of sub-profiling is the need for a large initial dataset to maintain adequate sample sizes down at sub-profile levels. Calculating secondary profiles can also result in a large number of sub-groups, and the clinical utility of the information gained by doing so is questionable. However, an approach that focusses on sub-profiles where there is significant variability in outcomes from the ‘primary’ profile should greatly reduce the number of sub-groups under consideration, and hence maintain the clinical utility of the overall profiling approach. In addition, where clinical utility is a secondary concern to the accurate prediction of an outcome, the probability of profile membership (posterior probability) could be used as a variable in a predictive model of outcome. Future research incorporating larger datasets could support these developments, and further explore the potential utility of posterior probabilities.

The identification of clinical profiles at baseline leads to questions about changes over time and trajectories of symptom change. What the profiles are able to tell us is that there are different groups of people at the point of their pre-treatment assessments, and we have shown that these groupings can be useful to predict treatment outcomes and potentially to inform discussions on treatment choice/selection. However, we do not know whether these groups continue to hold together all the way throughout treatment and whether the changes in symptoms during the course of treatment are similar between the profiles or whether trajectories of symptom change follow different courses within as well as between the profiles. If this were the case, it might mean that the profiles no longer hold together once treatment affects the factors which influenced profile membership. The use of outcome feedback tools in IAPT services has shown that when clinicians are informed about patients whose symptoms are following a course suggestive of no improvement or of deterioration in symptoms then this can improve patient outcomes by providing an opportunity to change treatments (Delgadillo et al., 2018). However, recent analysis of IAPT samples has suggested that a number of different courses of symptom change exist, including ones where response can be delayed until later in the course of treatment (Saunders et al., 2019), and it may be that certain clinical profiles are associated with later response, or that unique trajectories may exist within profiles.

The latent profile algorithm used in the current article uses nine routinely collected patient characteristics, available in all IAPT services in England and that could be collected in other types of treatment services. However, there are a number of other potential patient characteristics in addition to diagnosis that have been proposed as predictors of treatment outcome or the risk of relapse such as attentional control biases (Buckman, Underwood, et al., 2018), previous treatments (Cohen & DeRubeis, 2018), the expectation of treatment outcomes (Delgadillo et al., 2016), and the nature of the therapeutic alliance (Falkenström, Ekeblad, & Holmqvist, 2016). The inclusion of these factors as indicator variables in the latent profiles, or as potential moderators of treatment outcome, might potentially improve the ability to predict a variety of outcomes using profiling techniques.

### 6.3 Limitations

There are a number of limitations to the present analyses and profiling approach. Firstly, the dataset comes from the same two treatment services that provided the datasets for the development of the original algorithm. Although the present sample differed considerably from the original sample, the results of the present analyses suggest that the distribution and prediction of outcomes is robust over time in the services. A major limitation of the current profiling approach set out in this paper is the lack of data on diagnosis. Diagnosis is a major determinant of treatment in IAPT given the programme's link to NICE guidance (Clark, 2018). Inclusion of diagnoses in the profiles could improve the prognostic and predictive utility of the profiles but the advantage of the current model is that all data used to determine profile membership can be provided without the need for a clinical interview.

The current profiling system is also limited to only using the GAD-7 score as a measure of anxiety symptoms. IAPT services also collect anxiety disorder specific measures such as the Social Phobia Inventory (SPIN; Connor et al., 2000) for social phobia) which could be used to improve the predictive ability of the profiles. However, current use of these measures at initial assessment is limited in IAPT (Clark, 2018). A number of other potentially important patient level characteristics that have been associated with treatment outcome were not available in the current IAPT dataset, for example, chronicity of illness, relationship status, number of previous treatments and personality disorder comorbidity (DeRubeis et al., 2014). The inclusion of these variables in future profiling approaches might further improve their predictive ability.

Furthermore, the lack of randomisation in the routine dataset means that potential confounds in the allocation of patients to CBT versus counselling could not be controlled for. Although propensity score matching was used to reduce the effects of potential confounders, this did not include other potentially important characteristics that were not available in the dataset. The use of the propensity score matching may also have limited the observed benefits of HI over LI interventions, as some of the variation of membership to different profiles may have been associated with the allocation to HI over LI treatments (especially for profiles with increasing complexity). Further exploration of secondary profile membership could further inform differential treatment outcomes using the current profiling system.

## 7 Conclusions

Latent profile analysis and LVMM can provide a valuable method to predict a number of important outcomes from psychological treatment using routinely collected patient data. The eight-profile method discussed in this paper was used to predict the likelihood of reliable recovery and reliable improvement as well as deterioration as part of an out-of-sample validation study, with substantial variation in outcomes observed between profiles. In addition, a demonstration of matched controls within profiles comparing outcomes from High Intensity vs Low Intensity therapies and CBT vs Counselling (both HI therapies) suggests that the profiles can be further used to inform treatment selection decisions. Furthermore, the use of probabilistic functions in LVMM means that there is additional information beyond a patient's primary profile membership, and that there is potential to use information about the probability of membership to other, secondary profiles, to refine the prediction of treatment outcome.

## Declaration of competing interest

None to declare.
