# Supplementary material for: Latent variable mixture modelling and individual treatment prediction
Source: Behav Res Ther. 2020 Jan;124:103505. doi: 10.1016/j.brat.2019.103505 (PMC7417810; doi:10.1016/j.brat.2019.103505)
Supplement: Multimedia component 1 [file mmc1.pdf]

Supplementary materials to:

Latent variable mixture modelling and individual treatment prediction

* **Appendix A. Descriptives and distribution of profiles by financial year (page 2)**

* **Appendix B. Comparison of outcomes between profiles across all interventions (page 4).**

* **Appendix C. Proportion of patients reporting outcomes of interest by financial year (page 6)**

* **Appendix D. Comparison of outcomes by profiles for patients receiving LI vs HI psychological interventions (page 8)**

* **Appendix E. Comparison of outcomes by profiles for patients receiving CBT vs Counselling (page 10)**

Appendix A. Descriptives and distribution of profiles by financial year

Table A1. Descriptive statistics of the profiles on included indicator variables.

	Full sample	LP1 (17.6%)	LP2 (24.1%)	LP3 (3.04%)	LP4 (4.92%)	LP5 (9.58%)	LP6 (8.21%)	LP7 (9.64%)	LP8 (22.91%)
Age - Mean (SD)	37.56 (14.36)	32.67 (8.6)	29.49 (7.42)	67.85 (9.79)	66.07 (9.12)	55.02 (7.6)	39.99 (10.04)	44.03 (9.57)	28.78 (6.96)
PHQ-9 - Mean (SD)	13.8 (6.46)	5.71 (2.97)	11.41 (3.16)	4.94 (3.04)	11.09 (3.59)	18.07 (3.41)	13.66 (3.41)	23.18 (2.65)	18.98 (3.16)
GAD-7 - Mean (SD)	12.35 (5.43)	5.39 (2.63)	12.56 (3.08)	3.89 (2.49)	10.86 (3.33)	15.99 (2.87)	8.11 (2.64)	18.82 (2.25)	16.53 (2.83)
WSAS - Mean (SD)	17.17 (9.2)	8.88 (5.72)	14.66 (6.28)	6.24 (5.54)	12.01 (6.66)	18.6 (7.53)	20.96 (6.85)	31.09 (5.47)	22.49 (7.1)
Gender - n(%) female	29561 (67)	5023 (65)	7514 (71)	874 (66)	1536 (71)	2745 (65)	2044 (57)	2532 (60)	7293 (72)
Ethnic Group - n (%) Non-White	11242 (30)	1557 (24)	2097 (23)	151 (15)	277 (15)	934 (26)	1112 (36)	1503 (41)	3611 (41)
Medication prescribed - n (%) prescribed	21310 (51)	2818 (39)	3660 (37)	502 (41)	883 (45)	2502 (65)	2498 (72)	3228 (82)	5219 (54)
Welfare status - n (%) on benefits	11230 (26)	817 (11)	750 (7)	120 (9)	198 (10)	1840 (46)	1944 (54)	3181 (78)	2380 (24)
Phobia Self-rating - n (%) phobia	20992 (52)	1868 (25)	4366 (43)	239 (21)	697 (37)	2341 (64)	1792 (54)	3237 (93)	6452 (70)

Table A2. Distribution of patients entering treatment (1+ session) by profile & financial year.

Profile	1314		1415		1516		1617		1718		Total	
	n	%	n	%	n	%	n	%	n	%	n	%
LP1	720	16.35%	1,421	17.58%	1,847	18.32%	1,921	17.53%	1,993	17.51%	7,902	17.60%
LP2	889	20.19%	1,774	21.94%	2,479	24.59%	2,703	24.67%	2,975	26.14%	10,820	24.10%
LP3	139	3.16%	278	3.44%	377	3.74%	306	2.79%	265	2.33%	1,365	3.04%
LP4	189	4.29%	394	4.87%	529	5.25%	589	5.38%	509	4.47%	2,210	4.92%
LP5	434	9.86%	780	9.65%	971	9.63%	1,080	9.86%	1,039	9.13%	4,304	9.58%
LP6	479	10.88%	834	10.32%	814	8.07%	774	7.06%	787	6.92%	3,688	8.21%
LP7	534	12.13%	774	9.57%	933	9.26%	1,072	9.78%	1,017	8.94%	4,330	9.64%
LP8	1,019	23.14%	1,830	22.63%	2,131	21.14%	2,512	22.93%	2,794	24.55%	10,286	22.91%
Total	4,403		8,085		10,081		10,957		11,379		44,905	

Table A3. Distribution of patients included in outcome analyses by profile & financial year.

Profile	2013-14		2014-15		2015-16		2016-17		2017-18		Total	
	n	%	n	%	n	%	n	%	n	%	n	%
LP1	109	5.77%	229	6.97%	370	8.81%	362	8.34%	431	8.98%	1,501	8.11%
LP2	482	25.53%	924	28.11%	1,375	32.73%	1,524	35.12%	1,707	35.57%	6,012	32.47%
LP3	11	0.58%	26	0.79%	37	0.88%	21	0.48%	23	0.48%	118	0.64%
LP4	93	4.93%	194	5.90%	284	6.76%	285	6.57%	247	5.15%	1,103	5.96%
LP5	241	12.76%	368	11.20%	452	10.76%	433	9.98%	423	8.81%	1,917	10.35%
LP6	226	11.97%	405	12.32%	403	9.59%	346	7.97%	400	8.34%	1,780	9.61%
LP7	239	12.66%	296	9.01%	272	6.47%	277	6.38%	295	6.15%	1,379	7.45%
LP8	487	25.79%	845	25.71%	1,008	23.99%	1,091	25.14%	1,273	26.53%	4,704	25.41%
Total	1,888		3,287		4,201		4,339		4,799		18,514	

Figure A1. Distribution of profiles by financial year

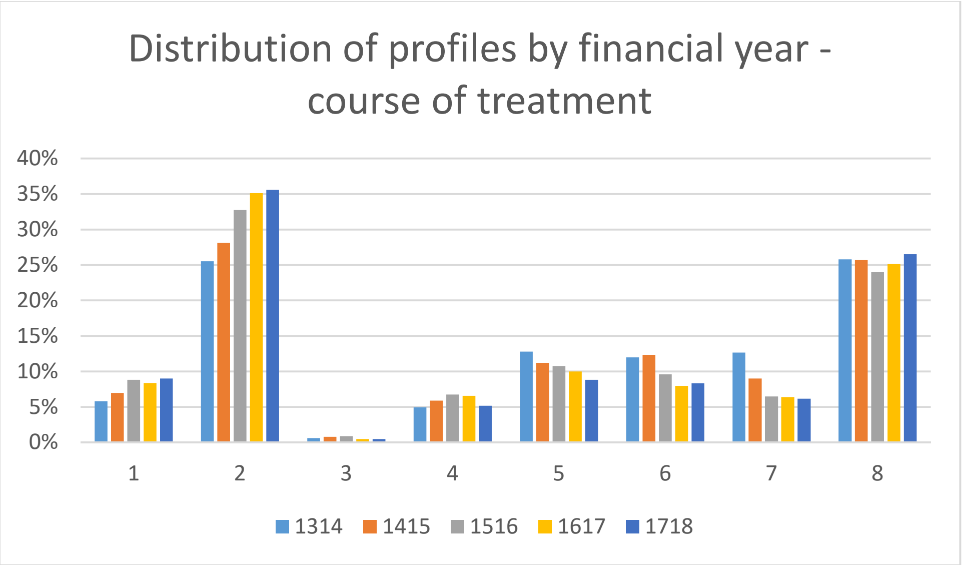

Appendix B. Comparison of outcomes between profiles (across all interventions).

Table B1. Proportion of patients meeting outcomes by profile

Profile	n	% Recovery	% Reliable Recovery	% Reliable Improvement	% Deterioration	% Attrition
LP1	1501	70.49%	48.26%	51.83%	9.33%	22.44%
LP2	6012	54.37%	51.39%	69.13%	7.52%	24.25%
LP3	118	76.27%	55.93%	58.12%	11.97%	14.42%
LP4	1103	63.92%	57.39%	68.27%	8.39%	12.82%
LP5	1917	32.24%	32.06%	67.98%	6.23%	21.10%
LP6	1780	53.03%	45.87%	56.75%	14.42%	25.90%
LP7	1379	17.69%	17.69%	57.39%	4.39%	33.00%
LP8	4704	31.97%	31.81%	70.33%	5.53%	34.97%
Total	18514	45.54%	41.49%	65.74%	7.54%	26.42%

Table B2. Logistic regression analyses comparing odds of recovery between all profiles and LP4, and all profiles against LP7.

Recovery									
	Odds ratio	p-value	95% Cis			Odds ratio	p-value	95% Cis	
LP4 = reference					LP7 = reference				
LP1	1.35	<0.001	1.14	1.59	LP1	11.11	<0.001	9.30	13.26
LP2	0.67	<0.001	0.59	0.77	LP2	5.54	<0.001	4.78	6.42
LP3	1.81	0.01	1.17	2.82	LP3	14.95	<0.001	9.57	23.36
LP5	0.27	<0.001	0.23	0.31	LP4	8.24	<0.001	6.85	9.91
LP6	0.64	<0.001	0.55	0.74	LP5	2.21	<0.001	1.87	2.62
LP7	0.12	<0.001	0.10	0.15	LP6	5.25	<0.001	4.45	6.21
LP8	0.27	<0.001	0.23	0.30	LP8	2.19	<0.001	1.88	2.54

Table B3. Logistic regression analyses comparing odds of reliable recovery between all profiles and LP4, and all profiles against LP7.

Reliable Recovery									
	Odds ratio	p-value	95% Cis			Odds ratio	p-value	95% Cis	
LP4 = reference					LP7 = reference				
LP1	0.69	<0.001	0.59	0.81	LP1	4.34	<0.001	3.65	5.15
LP2	0.78	<0.001	0.69	0.89	LP2	4.92	<0.001	4.24	5.70
LP3	0.94	0.761	0.64	1.38	LP3	5.90	<0.001	4.00	8.71
LP5	0.35	<0.001	0.30	0.41	LP4	6.26	<0.001	5.22	7.52
LP6	0.63	<0.001	0.54	0.73	LP5	2.20	<0.001	1.85	2.60
LP7	0.16	<0.001	0.13	0.19	LP6	3.94	<0.001	3.34	4.66
LP8	0.35	<0.001	0.30	0.40	LP8	2.17	<0.001	1.87	2.52

Table B4. Logistic regression analyses comparing odds of reliable improvement between all profiles and LP8, and all profiles against LP1.

Reliable improvement									
	Odds ratio	p-value	95% Cis			Odds ratio	p-value	95% Cis	
LP8 = reference					LP1 = reference				
LP1	0.45	<0.001	0.40	0.51	LP2	2.08	<0.001	1.85	2.34
LP2	0.94	0.180	0.87	1.03	LP3	1.29	0.19	0.88	1.89
LP3	0.59	0.005	0.40	0.85	LP4	2.00	<0.001	1.70	2.36
LP4	0.91	0.180	0.79	1.05	LP5	1.97	<0.001	1.71	2.27
LP5	0.90	0.059	0.80	1.00	LP6	1.22	0.01	1.06	1.40
LP6	0.55	<0.001	0.49	0.62	LP7	1.25	<0.001	1.08	1.45
LP7	0.57	<0.001	0.50	0.64	LP8	2.20	<0.001	1.95	2.49

Table B5. Logistic regression analyses comparing odds of deterioration b between all profiles and LP7, and all profiles against LP6.

Deterioration									
	Odds ratio	p-value	95% Cis			Odds ratio	p-value	95% Cis	
LP7 = reference					LP6 = reference				
LP1	2.24	<0.001	1.64	3.07	LP1	0.61	<0.001	0.49	0.76
LP2	1.77	<0.001	1.34	2.34	LP2	0.48	<0.001	0.41	0.57
LP3	2.96	0.001	1.60	5.48	LP3	0.81	0.463	0.45	1.43
LP4	2.00	<0.001	1.43	2.79	LP4	0.54	<0.001	0.42	0.70
LP5	1.45	0.023	1.05	1.99	LP5	0.39	<0.001	0.31	0.50
LP6	3.67	<0.001	2.75	4.91	LP7	0.27	<0.001	0.20	0.36
LP8	1.28	0.096	0.96	1.70	LP8	0.35	<0.001	0.29	0.42

Table B6. Logistic regression analyses comparing odds of attrition between all profiles and LP4, and all profiles against LP8.

Attrition									
	Odds ratio	p-value	95% Cis			Odds ratio	p-value	95% Cis	
LP4 = reference					LP8 = reference				
LP1	1.97	<0.001	1.57	2.47	LP1	0.54	<0.001	0.47	0.62
LP2	2.18	<0.001	1.79	2.65	LP2	0.60	<0.001	0.54	0.65
LP3	1.15	0.643	0.64	2.04	LP3	0.31	<0.001	0.18	0.54
LP5	1.82	<0.001	1.46	2.27	LP4	0.27	<0.001	0.22	0.33
LP6	2.38	<0.001	1.91	2.96	LP5	0.50	<0.001	0.43	0.57
LP7	3.35	<0.001	2.67	4.20	LP6	0.65	<0.001	0.57	0.74
LP8	3.66	<0.001	3.00	4.46	LP7	0.92	0.227	0.79	1.06

Appendix C. Proportion of patients reporting outcomes of interest by financial year.

Figure C1. Proportion of patients in recovery across profiles by financial year.

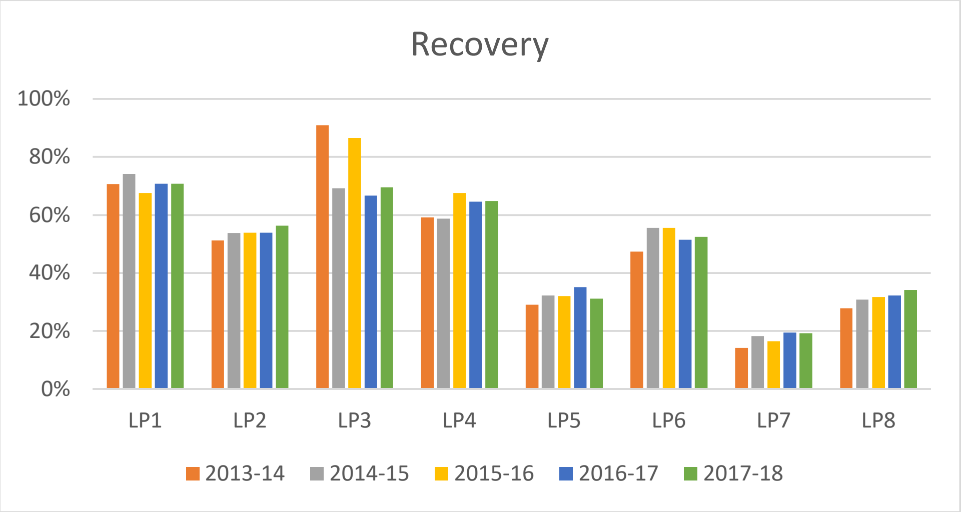

Figure C2. Proportion of patients reporting reliable recovery across profiles by financial year.

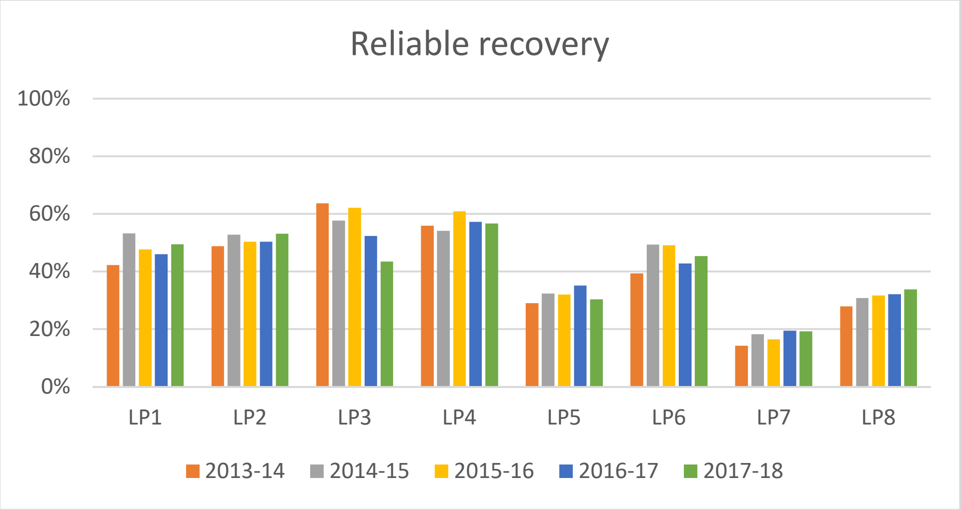

Figure C3. Proportion of patients reporting reliable improvement across profiles by financial year.

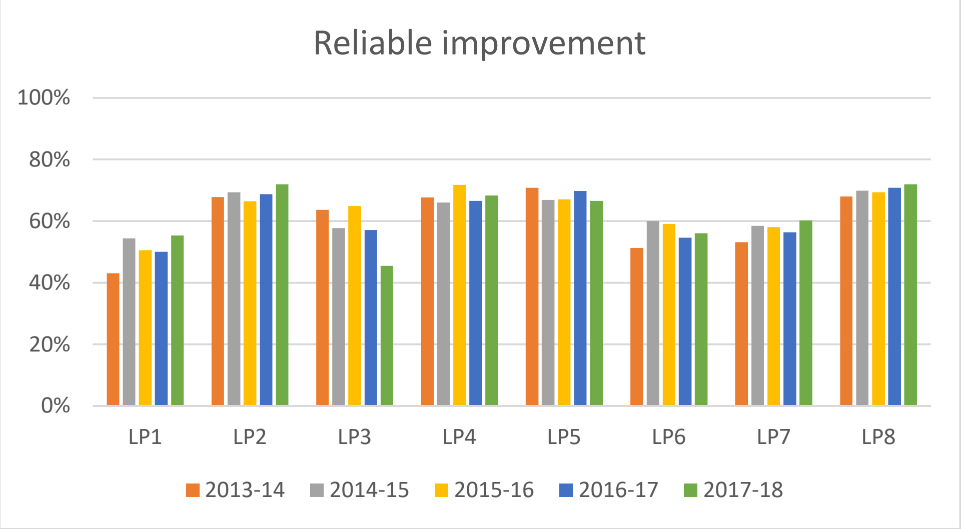

Figure C4. Proportion of patients reporting deterioration across profiles by financial year.

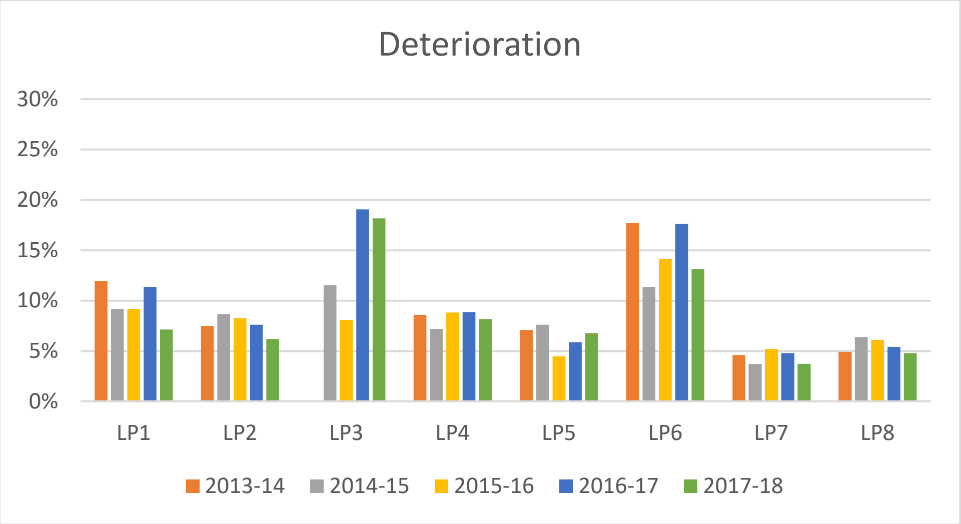

Figure C5. Proportion of patients dropping out across profiles by financial year.

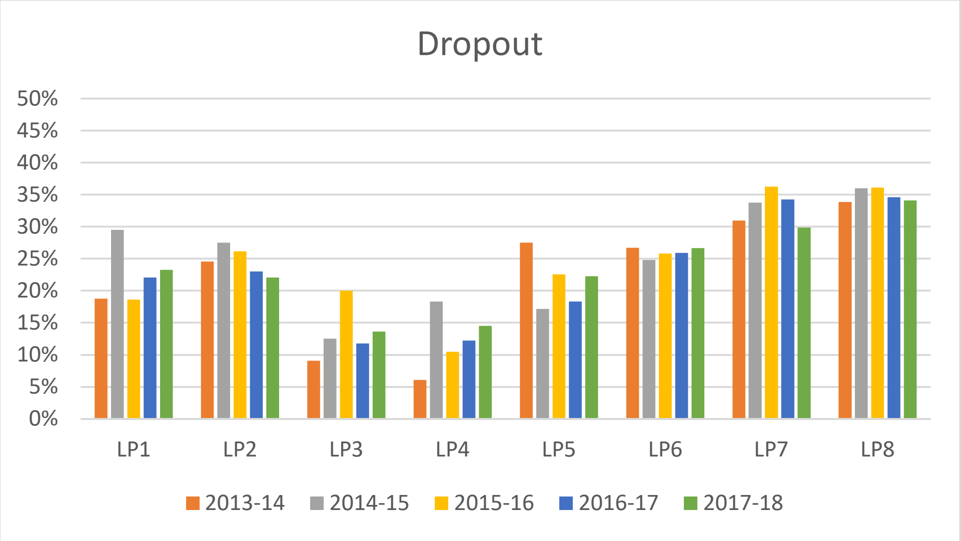

Appendix D. Comparison of outcomes by profiles for patients receiving LI vs HI psychological interventions.

Table D1: Number of patients receiving intensity of IAPT treatment by profile.

Profile	LI only		HI only		Received both		
	n	%	N	%	n	%	Total
LP1	304	20.25%	510	33.98%	687	45.77%	1501
LP2	1283	21.34%	1968	32.73%	2761	45.92%	6012
LP3	33	27.97%	50	42.37%	35	29.66%	118
LP4	228	20.67%	468	42.43%	407	36.90%	1103
LP5	408	21.28%	766	39.96%	743	38.76%	1917
LP6	444	24.94%	613	34.44%	723	40.62%	1780
LP7	214	15.52%	642	46.56%	523	37.93%	1379
LP8	926	19.69%	1716	36.48%	2062	43.84%	4704
Total	3840	20.74%	6733	36.37%	7941	42.89%	18514

Table D2. Proportion of patients reporting recovery following LI or HI interventions

	LI			HI			*Comparison*			
Profile	Recovered	Total	%	Recovered	Total	%	*Odds Ratio HI vs LI*	*Lower 95% CI*	*Upper 95% CI*	*p-value*
LP1	328	478	68.62%	338	476	71.01%	*1.120*	*0.849*	*1.477*	*0.422*
LP2	993	1903	52.18%	1025	1900	53.95%	*1.074*	*0.945*	*1.219*	*0.275*
LP3	8	11	72.73%	8	11	72.73%	*1.000*	*0.153*	*6.531*	*1.000*
LP4	241	366	65.85%	235	366	64.21%	*0.930*	*0.687*	*1.261*	*0.642*
LP5	211	701	30.10%	212	701	30.24%	*1.007*	*0.801*	*1.265*	*0.954*
LP6	290	580	50.00%	293	580	50.52%	*1.021*	*0.811*	*1.285*	*0.86*
LP7	64	571	11.21%	91	571	15.94%	*1.502*	*1.066*	*2.117*	*0.02*
LP8	395	1608	24.56%	493	1608	30.66%	*1.358*	*1.162*	*1.586*	*<0.001*

Table D3. Proportion of patients reporting reliable recovery following LI or HI interventions

	LI			HI			*Comparison*			
Profile	Reliable recovery	Total	%	Reliable recovery	Total	%	*Odds Ratio HI vs LI*	*Lower 95% CI*	*Upper 95% CI*	*p-value*
LP1	206	478	43.10%	225	471	47.77%	*1.208*	*0.935*	*1.560*	*0.148*
LP2	941	1903	49.45%	942	1882	50.05%	*1.024*	*0.902*	*1.164*	*0.71*
LP3	8	11	72.73%	6	11	54.55%	*0.450*	*0.076*	*2.669*	*0.379*
LP4	231	366	63.11%	210	366	57.38%	*0.787*	*0.585*	*1.058*	*0.113*
LP5	211	701	30.10%	208	697	29.84%	*0.988*	*0.786*	*1.242*	*0.916*
LP6	261	580	45.00%	250	580	43.10%	*0.926*	*0.734*	*1.168*	*0.515*
LP7	64	571	11.21%	91	571	15.94%	*1.502*	*1.066*	*2.117*	*0.02*
LP8	395	1608	24.56%	484	1599	30.27%	*1.333*	*1.141*	*1.558*	*<0.001*

Table D4. Proportion of patients reporting reliable improvement following LI or HI interventions

	LI			HI			*Comparison*			
Profile	Reliable improvement	Total	%	Reliable improvement	Total	%	*Odds Ratio HI vs LI*	*Lower 95% CI*	*Upper 95% CI*	*p-value*
LP1	208	478	43.51%	238	458	51.97%	*1.404*	*1.086*	*1.817*	*0.01*
LP2	1242	1886	65.85%	1274	1864	68.35%	*1.120*	*0.977*	*1.283*	*0.104*
LP3	8	11	72.73%	6	11	54.55%	*0.450*	*0.076*	*2.669*	*0.379*
LP4	281	366	76.78%	244	366	66.67%	*0.605*	*0.437*	*0.838*	*0.002*
LP5	474	701	67.62%	463	698	66.33%	*0.944*	*0.755*	*1.179*	*0.609*
LP6	326	580	56.21%	311	578	53.81%	*0.908*	*0.720*	*1.144*	*0.412*
LP7	268	571	46.94%	327	570	57.37%	*1.521*	*1.204*	*1.922*	*<0.001*
LP8	1112	1608	69.15%	1069	1596	66.98%	*0.905*	*0.780*	*1.050*	*0.187*

Table D5. Proportion of patients reporting clinical deterioration following LI or HI interventions

	LI			HI			*Comparison*			
Profile	Deterioration	Total	%	Deterioration	Total	%	*Odds Ratio HI vs LI*	*Lower 95% CI*	*Upper 95% CI*	*p-value*
LP1	43	478	9.00%	41	456	8.99%	*0.999*	*0.638*	*1.565*	*0.998*
LP2	139	1885	7.37%	137	1839	7.45%	*1.011*	*0.791*	*1.292*	*0.93*
LP3	0	11	0.00%	2	11	18.18%	*n/a*	*n/a*	*n/a*	*n/a*
LP4	31	366	8.47%	35	364	9.62%	*1.150*	*0.693*	*1.908*	*0.59*
LP5	55	701	7.85%	48	691	6.95%	*0.877*	*0.586*	*1.311*	*0.522*
LP6	103	580	17.76%	79	578	13.67%	*0.733*	*0.533*	*1.009*	*0.056*
LP7	26	571	4.55%	26	566	4.59%	*1.009*	*0.579*	*1.761*	*0.974*
LP8	101	1608	6.28%	86	1575	5.46%	*0.862*	*0.641*	*1.159*	*0.325*

Appendix E. Comparison of outcomes by profiles for patients receiving CBT vs Counselling.

Note: LP3 has been excluded from analyses due to small sample size.

Table E1. Proportion of patients reporting recovery following CBT or Counselling

	CBT			Counselling			*Comparison*			
Profile	Recovered	Total	%	Recovered	Total	%	*Odds Ratio Counselling vs CBT*	*Lower 95% CI*	*Upper 95% CI*	*p-value*
LP1	40	54	74.07%	42	54	77.78%	*1.225*	*0.506*	*2.966*	*0.653*
LP2	188	311	60.45%	176	312	56.41%	*0.847*	*0.615*	*1.165*	*0.306*
LP4	140	178	78.65%	104	178	58.43%	*0.381*	*0.239*	*0.608*	*<0.001*
LP5	103	271	38.01%	67	271	24.72%	*0.536*	*0.370*	*0.775*	*0.001*
LP6	46	112	41.07%	55	112	49.11%	*1.384*	*0.816*	*2.348*	*0.227*
LP7	35	171	20.47%	25	171	14.62%	*0.665*	*0.379*	*1.169*	*0.157*
LP8	104	252	41.27%	77	252	30.56%	*0.626*	*0.434*	*0.904*	*0.012*

Table E2. Proportion of patients reporting reliable recovery following CBT or Counselling

	CBT			Counselling			*Comparison*			
Profile	Reliable recovery	Total	%	Reliable recovery	Total	%	*Odds Ratio Counselling vs CBT*	*Lower 95% CI*	*Upper 95% CI*	*p-value*
LP1	27	53	50.94%	30	54	55.56%	*1.204*	*0.563*	*2.575*	*0.633*
LP2	168	308	54.55%	169	312	54.17%	*0.985*	*0.718*	*1.351*	*0.925*
LP4	124	178	69.66%	95	178	53.37%	*0.498*	*0.323*	*0.770*	*0.002*
LP5	98	266	36.84%	67	271	24.72%	*0.563*	*0.388*	*0.817*	*0.002*
LP6	44	112	39.29%	50	112	44.64%	*1.246*	*0.732*	*2.121*	*0.417*
LP7	35	171	20.47%	25	171	14.62%	*0.665*	*0.379*	*1.169*	*0.157*
LP8	102	250	40.80%	77	252	30.56%	*0.638*	*0.442*	*0.922*	*0.017*

Table E3. Proportion of patients reporting reliable improvement following CBT or Counselling

	CBT			Counselling			*Comparison*			
Profile	Reliable improvement	Total	%	Reliable improvement	Total	%	*Odds Ratio Counselling vs CBT*	*Lower 95% CI*	*Upper 95% CI*	*p-value*
LP1	29	53	54.72%	31	54	57.41%	*1.115*	*0.520*	*2.394*	*0.779*
LP2	217	302	71.85%	222	312	71.15%	*0.966*	*0.681*	*1.372*	*0.848*
LP4	133	178	74.72%	114	178	64.04%	*0.603*	*0.382*	*0.951*	*0.03*
LP5	207	271	76.38%	167	271	61.62%	*0.496*	*0.342*	*0.720*	*<0.001*
LP6	65	112	58.04%	64	112	57.14%	*0.964*	*0.567*	*1.638*	*0.892*
LP7	108	170	63.53%	112	171	65.50%	*1.090*	*0.699*	*1.698*	*0.704*
LP8	177	249	71.08%	168	252	66.67%	*0.814*	*0.557*	*1.188*	*0.286*

Table E4. Proportion of patients reporting clinical deterioration following CBT or Counselling

	CBT			Counselling			*Comparison*			
Profile	Deterioration	Total	%	Deterioration	Total	%	*Odds Ratio Counselling vs CBT*	*Lower 95% CI*	*Upper 95% CI*	*p-value*
LP1	4	53	7.55%	7	54	12.96%	*1.824*	*0.501*	*6.641*	*0.362*
LP2	21	302	6.95%	24	312	7.69%	*1.115*	*0.607*	*2.049*	*0.726*
LP4	7	176	3.98%	20	178	11.24%	*3.056*	*1.258*	*7.424*	*0.014*
LP5	11	258	4.26%	22	271	8.12%	*1.984*	*0.942*	*4.178*	*0.071*
LP6	20	112	17.86%	18	112	16.07%	*0.881*	*0.438*	*1.771*	*0.722*
LP7	2	169	1.18%	6	171	3.51%	*3.036*	*0.604*	*15.262*	*0.178*
LP8	6	246	2.44%	13	252	5.16%	*2.176*	*0.813*	*5.819*	*0.121*
